# Plasma lacosamide monitoring in children with epilepsy: Focus on reference therapeutic range and influencing factors

**DOI:** 10.3389/fped.2022.949783

**Published:** 2022-09-07

**Authors:** Yue Li, Hong-Li Guo, Yuan-Yuan Zhang, Na Dong, Ya-Hui Hu, Jing chen, Xiao-Peng Lu, Feng Chen

**Affiliations:** ^1^Department of Pharmacy, Pharmaceutical Sciences Research Center, Children's Hospital of Nanjing Medical University, Nanjing, China; ^2^Institute of Pharmaceutical Sciences, China Pharmaceutical University, Nanjing, China; ^3^Department of Neurology, Children's Hospital of Nanjing Medical University, Nanjing, China

**Keywords:** focal epilepsy, lacosamide, children, therapeutic drug monitoring, *C*_0_/Dose ratio

## Abstract

**Background:**

Lacosamide (LCM) is a newer anti-seizure medication (ASM) that was approved in China in 2018, but its real-world clinical data and plasma concentrations in Chinese children with epilepsy are very limited. Of note, the reference range for routine LCM therapeutic drug monitoring is still unknown. The purpose of this study was to investigate the efficacy and safety of LCM as a monotherapy or an adjunctive treatment with other ASMs and to evaluate the potential factors affecting its efficacy and variable LCM plasma concentrations in Chinese children with epilepsy.

**Methods:**

Children with epilepsy (<18 years) with routine plasma LCM monitoring from March 2019 to December 2021 at the Department of Pharmacy, Children's Hospital of Nanjing Medical University were retrospectively collected. Clinical data were obtained from the hospital information system.

**Results:**

76 pediatric patients (52 males) were finally enrolled. Mean age was 7.9 years (1.3–17.3 years) with a mean dose of LCM 6.3 mg/kg/day (2.0–11.3 mg/kg/day). The TDM data as a whole showed that the median plasma trough concentration (*C*_0_) was 3.42 μg/mL (1.25–8.31 μg/mL). A 6-month LCM add-on therapy produced 70% of patients achieving ≥50% seizure frequency reductions, and the number was 81% for the one-year follow-up findings. Interestingly, more patients who took LCM monotherapy achieved seizure freedom over the same periods of follow-up observations. Under maintenance dosages, approximately 92.1% of the *C*_0_ values were 2.0–7.0 μg/mL. The plasma-*C*_0_-to-daily dose (*C*_0_/Dose) ratio was significantly associated with age and body weight (BW). The *C*_0_/Dose ratio in patients aged 1– ≤ 6 and 6– ≤ 12 years was significantly higher by 81% and 29% than those aged 12– ≤ 18 years, respectively. The *C*_0_/Dose ratio in patients with a BW of ≥40 kg was 1.7-fold lower than in patients with a BW of ≤ 20 kg. In addition, complex LCM-ASMs interactions were observed. Oxcarbazepine significantly decreased the *C*_0_/Dose ratio of LCM by 28%.

**Conclusion:**

This retrospective study confirmed the effectiveness and tolerability of the LCM treatment used alone or with other ASMs in children with focal epilepsy. Children with higher BW and older age have lower *C*_0_/Dose ratio. Complex drug interactions between LCM and other concomitant ASMs were revealed. Notably, based on the data in our hands, the reference range, *i.e*., 2.0–7.0 μg/mL, for routine LCM monitoring may be feasible. The real-world evidence of this study supports LCM as a promising option in children with focal epilepsy.

## Introduction

China has ~10 million people with epilepsy (1), and around two-thirds of people are under 18 years of age (2). Childhood epilepsies present broad management challenges that are unique to this age group. These challenges mainly include the precision diagnoses; the therapy options; the developmental, cognitive, and behavioral comorbidities of epilepsy; and the likelihood that those different factors interact with developmental processes in the young brain (3). Nearly 20 different anti-seizure medications (ASMs) and non-pharmacological options are now available in China, but there are still unmet needs for epilepsy management (1), with therapeutic aims not only to achieve overt freedom from seizures, but also to actively abolish abnormal electrical activity in the developing brain.

With new-generation ASMs, such as lacosamide (LCM), seizure control with less side effects and food- and/or drug-drug interactions is expected, in an attempt to target the causes and mechanisms of epilepsy rather than its symptoms (4). LCM, the R-enantiomer of 2-acetamido-N-benzyl-3-methoxypropionamide, is a functionalized amino acid analog of D-serine. LCM exerts distinct mechanisms of action over other ASMs by selectively changing voltage-gated sodium channel into a slow and inactivated state, resulting in stabilization of hyperexcitable neuronal membranes; and by binding to the collapsin response mediator protein 2, which plays critical roles in the process of neuronal differentiation, growth, polarization, control of axonal outgrowth and probably also epileptogenesis (5, 6). These unique properties lead to its powerful anti-seizure effects while retaining normal brain functions.

The US FDA approved LCM as an adjunctive therapy for partial-onset (focal) seizures in October 2008 (5). Nowadays, as a prescription medicine, LCM is used to treat focal-onset seizures in people 1 month of age and older or used with other medicines to treat primary generalized tonic-clonic seizures in people 4 years of age and older, according to the revised version of package insert in 2021. Nevertheless, it is unclear if LCM is safe and effective for partial-onset seizures in children under 1 month of age or for primary generalized tonic-clonic seizures in children under 4 years of age. Recent study revealed that LCM might also be useful as the first-line monotherapy for adults with newly diagnosed epilepsy (7). In China, LCM was approved in 2018 as an adjunctive therapy to treat focal-onset seizures in people 4 years of age and older. However, the safety and effectiveness profiles of LCM stay understudied in Chinese pediatric patients. The approval was granted mainly based on the extrapolation of efficacy and safety data from those western pediatric subjects (8). Therefore, the efficacy, tolerability, and pharmacokinetics of LCM are worthy of further validation by enrolling both RCTs and real-world observational studies with different time-period treatments (9).

A long-term, open-label extension of a randomized, controlled trial revealed that LCM was well-tolerated as long-term adjunctive therapy in Chinese adults with epilepsy and uncontrolled focal seizures, with improvements in seizure reduction maintained over 36 months of treatment (10, 11). Notably, a retrospective study of 72 pediatric patients with epilepsy in Uygur, China, showed that LCM therapy is safe and effective for epilepsy in children, resulting in a reduction in the seizure frequency (8). However, there is limited data on the effectiveness and safety of LCM in Han Chinese children with epilepsy.

On the other hand, it is evident that the systemic exposure to LCM depends partly on age and sex, thereby requiring pharmacokinetic monitoring to define the optimal dosage that guarantees therapeutic efficacy with tolerable side effects (4). Interestingly, Zhao et al. (12) found that *ABCB1* polymorphisms might affect LCM serum concentrations and treatment efficacy in Uygur pediatric patients with epilepsy, leading to drug resistance. In addition, evidence of drug-drug interactions also justifies monitoring epileptic patients taking LCM (13). In a sense, LCM monitoring demands sensitive and robust bioanalytical techniques that guarantee an accurate LCM measurement in plasma or serum. Moreover, it is also meaningful to define a specific reference range of LCM for Chinese people, especially for pediatric patients. Although several ranges have been recommended, the optimal therapeutic range is still inconclusive for those western populations (14).

This retrospective study aimed to (1) review the efficacy and safety of LCM as a monotherapy or an adjunctive treatment with other ASMs in Chinese children with epilepsy; (2) identify the potential factors affecting its plasma concentrations; and (3) suggest a specific plasma reference range for LCM.

## Patients and methods

### Patients

**Figure 1:**
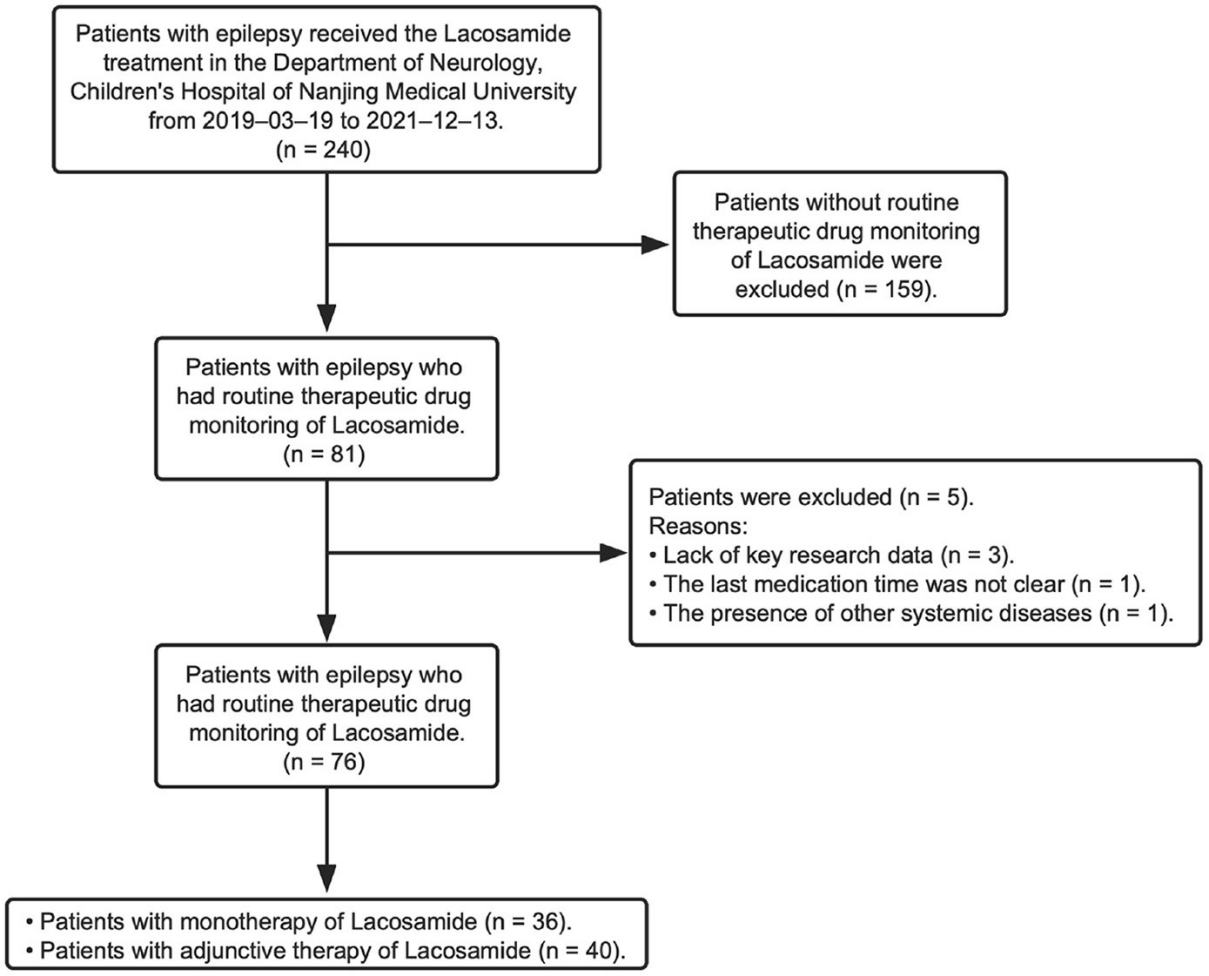
Numbers of patients who were eligible for this study.

This study retrospectively reviewed children (<18 years) who were diagnosed with epilepsy and did the LCM treatment at the Children's Hospital of Nanjing Medical University from March 2019 to December 2021 (Figure 1). Diagnosis of epileptic seizures and syndromes was based on the Classification of Epileptic Seizures (15), after reviewing the semiology of seizures, electroencephalography (EEG), and magnetic resonance imaging (MRI) findings. Patients were excluded from the study if: (1) they received LCM treatment but did not have routine LCM concentration monitoring data; (2) if they had an underlying metabolic and systemic disorder; (3) if their detailed information was absent in the hospital information system (HIS). The Ethics Committee of the Children's Hospital of Nanjing Medical University granted the ethical approval for the study (Protocol number 202204021-1). Written consents were waivered due to the retrospective nature of the study.

### Treatment protocol

All patients included in this study received oral LCM as monotherapy or add-on therapy.

For children aged 4 to 17 years, the starting dose was 2 mg/kg/day, which was raised to an initial therapeutic dose of 4 mg/kg/day after 1 week. Based on clinical response and tolerability, the maintenance dose might be increased weekly by 2 mg/kg/day. Gradually titrate the dose until the best response was achieved. Of note, for children weighing ≥11 but <30 kg, due to the increased total clearance compared with adults, the maximum dose did not exceed 12 mg/kg/day, and the maintenance dose was 6 to 12 mg/kg/day. For children weighing ≥30, but <50 kg, the maximum dose was 8 mg/kg/day, and the maintenance dose was 4 to 8 mg/kg/day. The daily dose was taken in two divided doses. For children weighing ≥50 kg, the starting dose is 50 mg twice a day, then the dose was increased to an initial therapeutic dose of 100 mg twice a day, in the morning and evening, after 1 week.

For pediatric patients below the age of 4 years with focal epilepsy, the informed consent was obtained from a parent of each patient due to the off-label use nature. According to the package insert, the dose tailoring was performed based on the body weight (BW), *i.e*., the recommended dosages for weighing 6 kg to 11 kg and weighing <6 kg.

Collectively, the mean initial dose of LCM was 2.13 mg/kg/day, and the mean maintenance dose was 6.29 mg/kg/day. After three consecutive days of the administration, the trough concentration (*C*_0_) of LCM was measured by LC-MS/MS method before the morning dose, and then each patient was followed up periodically for monitoring efficacy, safety, LCM levels, and laboratory tests.

### Definitions of clinical response

Diagnosis of epileptic seizures and syndromes was based on the Classification of Epileptic Seizures (15), after reviewing the semiology of seizures, EEG, and MRI findings. The seizure frequency of 1 month before starting LCM therapy was set as a baseline value. To measure the curative effect of LCM therapy, the seizure frequency at 1, 3, 6, 12, and 24 months after starting LCM therapy were recorded.

The definitions for clinical response to the treatment as following were based on the seizure frequency compared with the baseline values: (1) seizure-freedom (SF), *i.e*., absence of seizures on unchanged medication; (2) seizure frequency reduction (SFR), *i.e*., patients with 50% or more reduction of baseline seizure frequency on unchanged medication; (3) ineffectiveness (IE), *i.e*., patients with <50% reduction in seizure frequency on unchanged medication. Accordingly, children with seizure freedom, ≥50% seizure reduction, and <50% seizure frequency induction for a period of at least 6 months were considered as complete responders, responders, and non-responders, respectively.

### Data collection

We collected various data on age, sex, BW, types of seizures, EEG findings, neuroimaging, duration of epilepsy before starting LCM therapy, duration of LCM treatment, number and type of previous ASMs treatment, concomitant ASMs used, treatment response, reported side effects. Specific data on LCM including its initial and maximal dose, and routine therapeutic drug monitoring if possible were also reviewed. The efficacy measures were analyzed based on the change in seizure frequency.

### Routine therapeutic monitoring of LCM

Whole blood samples are routinely transported to our lab for monitoring steady-state plasma LCM levels in pediatric patients with LCM monotherapy or adjunctive therapy. The bioanalysis was performed on an LC-MS/MS system. In brief, the LC-MS/MS system consisted of a Triple Quad^TM^ 4500MD mass spectrometer (AB Sciex Pte. Ltd, Singapore) interfaced *via* a Turbo V^TM^ ion source with a Jasper^TM^ liquid chromatography system (AB Sciex Pte. Ltd, Singapore), which comprises a binary pump (Sciex Dx^TM^), an online degasser (Sciex Dx^TM^), an autosampler (Sciex Dx^TM^), and a column oven (Sciex Dx^TM^). The AB-SCIEX Analyst software packages (version 1.6.3) were used to control the LC-MS/MS system, as well as for data acquisition and processing. The chromatographic separation was achieved on a Kinetex C18 column (2.1 x 50 mm, 5 μm, Phenomenex) with a security Guard-C18 column (4 x 2.0 mm, Phenomenex), pumped at a flow rate of 0.35 mL/min. Gradient elution was carried out with mobile phase A consisting of 0.008 mM FA in water and mobile phase B of MeOH containing the same FA level. The gradient elution program was as follows: 0–0.3 min, 1% B; 0.3–0.4 min, 1–20% B; 0.4–2.7 min, 20–50% B; 2.7–6.5 min, 50% B; 6.6–8.8 min, 100% B; 8.8–8.9 min, 100–1% B;8.9–10.0 min, 1% B. The column and auto-sampler were maintained at 30 and 4°C, respectively. MeOH precipitation was used for sample clean-up and the 5 μL supernatant was injected into LC-MS/MS for analysis. Ionization mode was ESI positive and two mass transitions (m/z 251.3 → 108.1 and 257.1 → 108.1) were monitored for LCM and its internal standard. LCM quantification was normalized by using stable-isotope-labeled LCM-d6. Collectively, no matrix effect or carryover was observed. The intra- and inter-day accuracy and precision of the assay were all acceptable according to US FDA guidance. The method development and validation data for simultaneous determination of 15 ASMs including LCM has been published elsewhere (16).

### Statistical analysis

All data were statistically analyzed using GraphPad Prism 9 (GraphPad Software, La Jolla, CA, United States) and SPSS version 26.0 software (IBM, Armonk, USA). Shapiro-Wilk tests were used to assess normality. Demographic data and clinical characteristics were described as the frequency for categorical variables, means and standard deviations for normally distributed continuous variables, and median with an interquartile range for non-normally distributed continuous variables, respectively. Continuous variables were compared using the Mann–Whitney U test. Differences between independent groups were assessed using the Kruskal–Wallis test and Dunn's test. Correlations were tested by Spearman's correlation coefficient analysis. A *P*-value of < 0.05 was considered statistically significant.

## Results

### Characteristics of pediatric patients

A total of 76 children (52 males) met the inclusion criteria (Table 1). 98.7% of patients were diagnosed with focal seizures. The median epilepsy duration from the time of first seizure was 18 months (IQR 31.5). The median number of ASMs used before starting LCM treatment was one (IQR 2), and the median number of concomitant ASMs after LCM therapy initiation was two (IQR 2) (Table 1).

**Table 1:** Clinical characteristics of patients.

**Characteristics**	**Value**
**Age (year)**
Mean ± SD	7.9 ± 3.5
Range	1.3–17.3
**Sex**
M	52
F	24
**Weight (kg)**
Range	10–75
**Type of seizures**, ***n*** **(%)**
Focal seizures	75 (98.7%)
Unknown	1 (1.3%)
**Dose (mg/kg)**
Mean ± SD	6.3 ± 1.9
Range	2.0–11.3
**Number of previous ASMs**
Median	1
IQR	2
**Number of ASMs when LCM initiated**, ***n*** **(%)**
0	36 (47.4%)
1	21 (27.6%)
2	15 (19.7%)
3	3 (3.9%)
4	1 (1.3%)
**Concomitant ASMs**, ***n*** **(%)**
VPA	24 (31.6%)
LEV	15 (19.7%)
OXC	11 (14.5%)
PER	6 (7.9%)
LMT	5 (6.5%)
CZP	3 (3.9%)
TPM	1 (1.3%)

M, male; F, female; ASM, antiseizure medicine; LCM, lacosamide; VPA, valproic acid; LEV, levetiracetam; OXC, oxcarbazepine; PER, perampanel; LMT, lamotrigine; CZP, clonazepam; TPM, topiramate.

LCM therapy was initiated when the patient was at a median age of 6.6 (IQR 5) years. Ten children were 4 years of age or younger, but none of them aged 1 month to 12 months. The median treatment duration was 5.5 months (IQR 9.4). Mean maintenance LCM dose was 6.3 mg/kg daily (range 2.0–11.3 mg/kg/day).

### Clinical outcomes

The seizure frequency at 1, 3, 6, 12, and 24 months after starting LCM therapy were recorded and compared with the baseline values (Tables 2, 3). Before starting LCM therapy, 92% (*n* = 70) of patients had experienced unsuccessful epilepsy control. Notably, over a follow-up period of 6 months, 15 and 10 patients became seizure free while receiving LCM as monotherapy and add-on therapy, respectively. Moreover, 6 more patients achieved on seizure reduction and only 9 patients (30%) were poorly responsive to the LCM adjunctive therapy. Collectively, a 6-month LCM add-on therapy produced complete or partial remission of 70% (*n* = 21) of patients, and the number was 81% (*n* = 17) for the 1-year follow-up findings. However, no any clinical improvement was noted in 3 of nine children (33.3%) after a 2-year follow-up with LCM add-on therapy (Table 2). Interestingly, more patients who took LCM monotherapy achieved on seizure freedom (*i.e*., higher remission rate) over a similar period of follow-up observation (Table 3).

**Table 2:** The seizure frequencies at 1, 3, 6, 12, and 24 months after starting LCM adjunctive therapy.

**Time (month)**	**IE**	**SFR**	**SF**
Baseline	34 (85%)	6 (15%)	0
1	12 (30.8%)	6 (15.4%)	21 (53.8%)
3	13 (37.1%)	4 (11.4%)	18 (51.4%)
6	9 (30%)	6 (20%)	15 (50%)
12	4 (19%)	4 (19%)	13 (62%)
24	3 (33.3%)	4 (44.4%)	2 (22.2%)

IE, ineffectiveness, *i.e*., patients with <50% reduction in seizure frequency on unchanged medication; SFR, seizure frequency reduction, *i.e*., patients with 50% or more reduction of baseline seizure frequency on unchanged medication; SF, seizure-freedom, *i.e*., absence of seizures on unchanged medication.

**Table 3:** The seizure frequencies at 1, 3, 6, 12, and 24 months after starting LCM monotherapy.

**Time (month)**	**IE**	**SFR**	**SF**
Baseline	36 (100%)	0	0
1	6 (16.7%)	2 (5.6%)	28 (77.7%)
3	1 (3.8%)	1 (3.8%)	24 (92.3%)
6	NA	NA	10 (100%)
12	NA	NA	4 (100%)
24	NA	NA	NA

IE, ineffectiveness, *i.e*., patients with <50% reduction in seizure frequency on unchanged medication; SFR, seizure frequency reduction, *i.e*., patients with 50% or more reduction of baseline seizure frequency on unchanged medication; SF, seizure-freedom, *i.e*., absence of seizures on unchanged medication; NA, not available.

In addition, all patients could tolerate the LCM medications. During the 2-year treatment period, 3 (3.9%) patients had dizziness; 1 (1.3%) had hypersomnia; 1 (1.3%) had diplopia and hypersomnia.

### Plasma *C*_0_ of LCM

**Figure 2:**
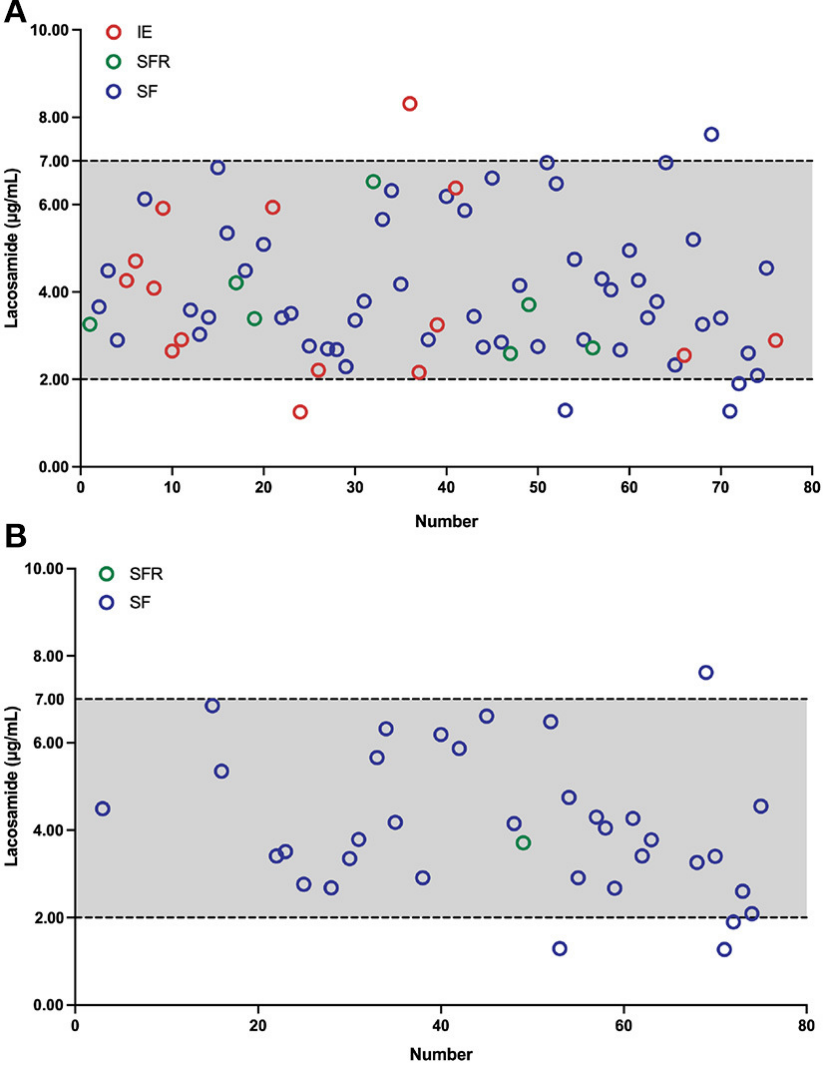
Plasma LCM concentrations *C*_0_ (μg/mL) in children with epilepsy, the x-axis shows the number of patients. Two dotted lines depict the measured *C*_0_ values in our findings. Different colored circles denote *C*_0_ measurements after the maintenance dosage. Red circles indicate *C*_0_ measurements of the ineffectiveness (IE) group, green circles represent *C*_0_ measurements of the seizure frequency reduction (SFR) group, and blue circles denote *C*_0_ measurements of the seizure-freedom (SF) group, respectively. **(A)** As an adjunctive therapy, LCM *C*_0_ (μg/mL) values in 76 children with epilepsy. **(B)** As a monotherapy, LCM *C*_0_ (μg/mL) measurements in 36 children with epilepsy.

The blood *C*_0_ was monitored throughout the entire treatment period. To avoid introducing bias from multiple samples from each individual patients, the first measure was used when more than one result was available. In total, 76 measurements were recorded for all the 76 patients, with *C*_0_ values found to be between 1.25 and 8.31 μg/mL (Figure 2A). Notably, approximately 92.1% of the monitored *C*_0_ values ranged from 2.0 to 7.0 μg/mL. Intriguingly, in the range of 2.0–7.0 μg/mL, 71.4% (*n* = 50) of patients were in the SF group, which demonstrated that most patients became seizure free after LCM treatment. When we fixed our eyes on the LCM monotherapy, 36 measurements were recorded. Moreover, approximately 88.8% of the *C*_0_ values scattered at 2.0–7.0 μg/mL and 96.9% (*n* = 31) of patients became seizure free (Figure 2B).

### Age, BW, sex and the *C*_0_/Dose ratio of LCM

**Figure 3:**
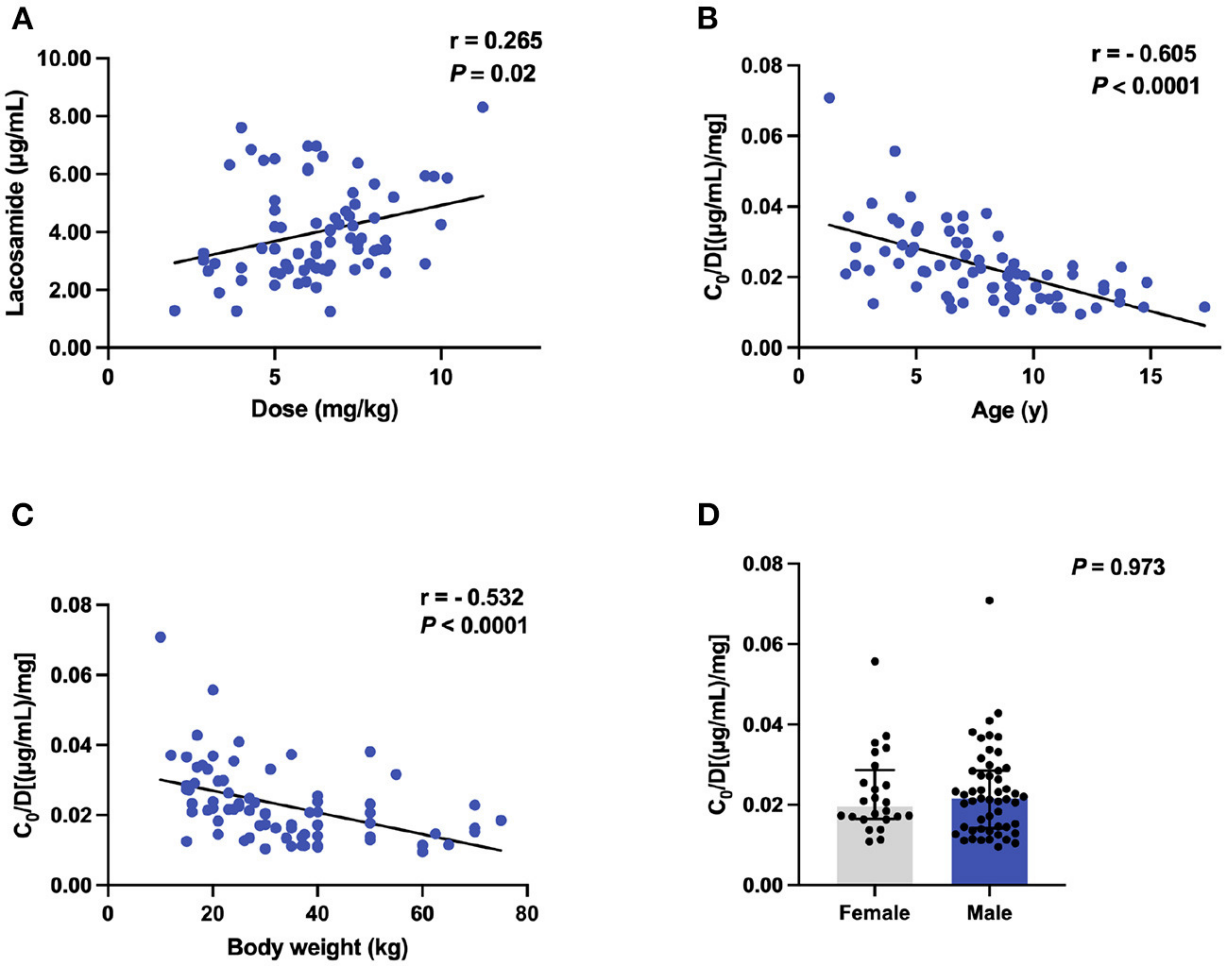
The *C*_0_ and *C*_0_/Dose ratio [(μg/mL)/mg] of LCM in polytherapy (*n* = 76). **(A)** Correlation between *C*_0_ and dose (mg/kg); **(B)** Correlation between *C*_0_/Dose ratio and ages; **(C)** Correlation between *C*_0_/Dose ratio and BW; **(D)** A comparison of *C*_0_/Dose ratio in both sexes.

We observed a weak positive correlation between monitored *C*_0_ values and LCM doses (r = 0.265, *P* = 0.02; Figure 3A) if we did not distinguish between monotherapy and combination therapy. In those patients, we found a significant negative correlation between age and *C*_0_/Dose ratio (r = − 0.605, *P* < 0.0001; Figure 3B). Specifically, the dose-corrected *C*_0_ values were significantly higher in children with 1– ≤ 6 (*n* = 23) and 6– ≤ 12 years of age (*n* = 43) than those patients with 12– ≤ 18 year of age (*n* = 10, *P* < 0.001) by 81 and 29%, respectively. Similarly, we also revealed a negative correlation between BW and *C*_0_/Dose ratio (r = - 0.532, *P* < 0.0001; Figure 3C), and the values in children with a BW of ≥40 kg were 1.7-fold and 1.2-fold lower than those in patients with a BW of ≤ 20 kg and between 20 to 40 kg, respectively (*P* < 0.001). In addition, no significant differences were found in *C*_0_/Dose ratio between individuals of both sexes (*P* = 0.973; Figure 3D). Nevertheless, males exposed to higher LCM levels.

**Figure 4:**
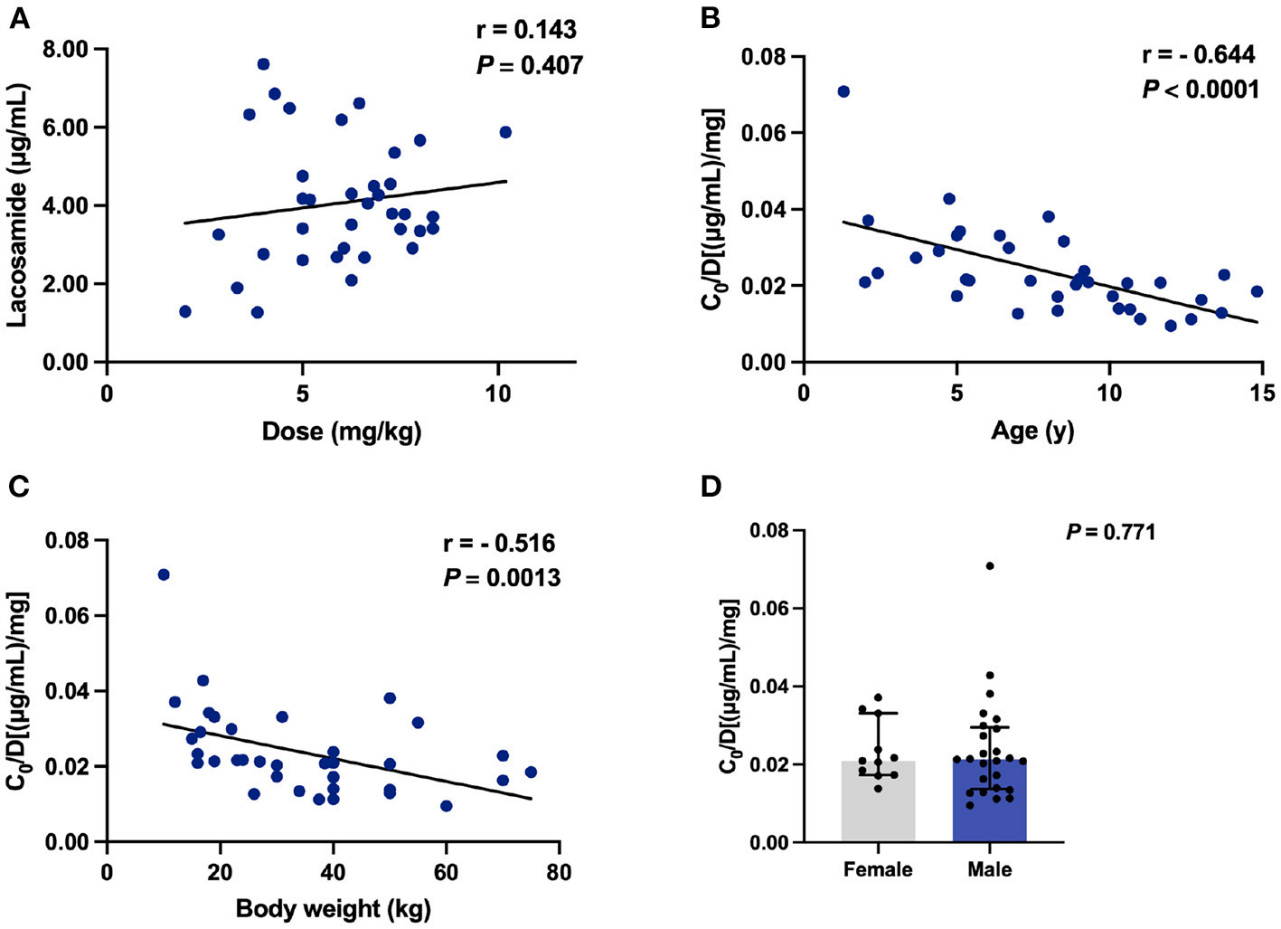
The *C*_0_ and *C*_0_/Dose ratio [(μg/mL)/mg] of LCM in monotherapy (*n* = 36). **(A)** Correlation between *C*_0_ and dose (mg/kg); **(B)** Correlation between *C*_0_/Dose ratio and ages; **(C)** Correlation between *C*_0_/Dose ratio and BW; **(D)** A comparison of *C*_0_/Dose ratio in both sexes.

We next tested whether the above-mentioned findings were still retained when the adjunctive therapy data were removed. No correlation was found between *C*_0_ values and doses of LCM (r = 0.143, *P* = 0.407; Figure 4A). Notably, the significant negative correlation between age and *C*_0_/Dose ratio (r = - 0.644, *P* < 0.0001; Figure 4B) could still be observed. The same was true for BW and *C*_0_/Dose ratio (r = - 0.516, *P* = 0.0013; Figure 4C).

### Concomitant drugs and the *C*_0_/dose ratio of LCM

**Figure 5:**
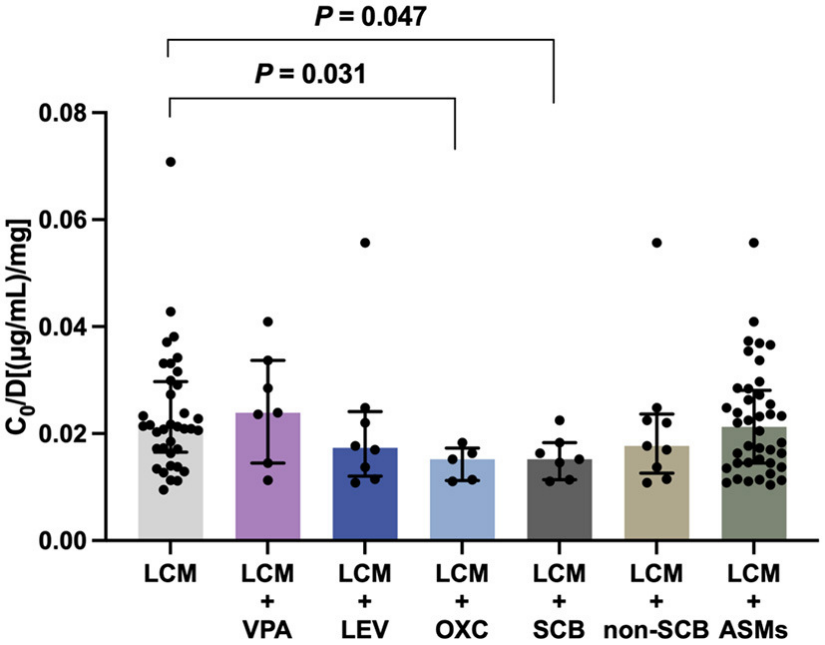
The *C*_0_/Dose ratio [(μg/mL)/mg] of LCM in polytherapy. A comparison of *C*_0_/Dose ratio between monotherapy and coadministration with valproic acid (VPA), levetiracetam (LEV), oxcarbazepine (OXC), sodium channel blocking (SCB) agents and non-SCB medications.

To test whether coadministration contributes to the *C*_0_/Dose ratio, we evaluated the influences of various concomitant therapies on plasma LCM levels. Notably, oxcarbazepine (OXC), but not valproic acid (VPA) or levetiracetam (LEV), significantly decreased the *C*_0_/Dose ratio of LCM by 28% (*P* = 0.031; Figure 5). Interestingly, the coadministration with other ASMs did not put any impact on the *C*_0_/Dose ratio of LCM (*i.e*., LCM + ASMs *vs*. LCM).

We next evaluated the potential influences of concomitant ASMs with different mechanisms of action, including sodium channel blocking (SCB) agents [*i.e*., OXC, lamotrigine (LMT), and topiramate (TPM); LCM + SCBs, *n* = 7], and non-SCB medications [*i.e*., levetiracetam (LEV), perampanel (PER), or clonazepam (CZP); LCM + non-SCB, *n* = 9]. Of note, the add-on SCB medications (*P* = 0.047), predominantly OXC, significantly decreased the *C*_0_/Dose ratio of LCM (Figure 5).

### Other factors affecting the *C*_0_/dose ratio of LCM

Since epilepsy is a chronic disease, we wondered if some other factors play potential roles. To test this, we evaluated the duration of LCM treatment, disease duration, MRI and EEG readings in these pediatric patients. Only negligible effects were observed. Interestingly, there were no any statistically significant differences between complete responders, responders, and non-responders groups regarding *C*_0_ and *C*_0_/Dose ratio.

## Discussion

Seizures pose risks, especially for pediatric patients, and the main goal of pharmacological treatment for epilepsy is to eliminate seizures completely while minimizing the adverse effects of ASMs, or no adverse events. To achieve this, drug dosages need to be individualized. In fact, the clinical use of a new ASM follows a stepwise vigorous approach to better understand its mechanism of action, efficacy, pharmacokinetics, and tolerability. LCM gained its approval from China government in 2018, but reports on its clinical experience with LCM as an add-on or first-line monotherapy in Chinese children, even in adults, with epilepsy are still rare (8–11, 17). No or only sparse data on LCM concentration monitoring at this young age group are available. Therefore, real-world data collected during routine clinical care for young pediatric patients have received increasing attention as a source of valuable information to support dosage optimization. This retrospective study assessed the efficacy of LCM as mono- and add-on therapy in a Chinese pediatric population. Specifically, in the current study, we explored for the first time the effect of demographic and clinical variables on the LCM plasma concentration in children with focal epilepsy in our clinical practice.

In this study, adjunctive therapy of LCM was shown to be effective and safe with as many as approximately 81 and 67% of children experiencing ≥50% seizure frequency reduction by the end of a 1- and 2-year follow-up period, respectively (Table 2). For LCM monotherapy, 10 and 4 patients completed a 6- and 12-months follow-up observation, respectively, and all of them became seizure free (Table 3). One very recent retrospective study of pediatric patients with epilepsy in the Uygur area of China has revealed that the addition of LCM to antiseizure therapy resulted in a positive response in approximately 69% of children over a minimum 1-year follow-up period (8). Some retrospective studies in pediatric patients of other nations so fat have shown that the proportion of responders varies between ~30 and 70% (18–24). Collectively, our efficacy findings are overall comparable to those previously reported from Western and Asian countries.

A major finding of this study was that a reference therapeutic *C*_0_ range of LCM (*i.e*., 2.0–7.0 μg/mL) (Figure 2) was established to match the efficacy and tolerability seen in our pediatric patients. Therapeutic drug monitoring (TDM) of ASMs assists in guiding and tailoring ASM therapy, while also avoiding potential associated toxicity in routine clinical practice, because the clinical response has been shown to correlate better with the drug concentration than the dose (25). In fact, TDM requirement whether or not and the reference range for LCM monitoring in children and adults are still controversial (14). Previously, 2.5–10 μg/mL has been suggested as a target range, but those values were partly derived from non-drug-fasting blood samples (26). Burns et al. (27) revealed that 94% of patients had serum concentrations in the reference range of 3–10 μg/mL in Norway, and a similar reference range of 2.25–8.75 μg/mL is used in Denmark. However, Perrenoud et al. (28) concluded that the reference range of 10–20 μg/mL was more effective in reducing seizures. But to emphasize again, the reference range for LCM monitoring in Chinese children is not available. In our study, under maintenance dosages, approximately 92.1% of the *C*_0_ values varied from 2.0 to 7.0 μg/mL and the matched mean daily dose was 6.38 mg/kg (range 2.86–10.19 mg/kg/day). More than 81.4% of *C*_0_ values in children achieved on >50% seizure frequency reduction (Figure 2A). Similar findings (*i.e*., 2.0–7.0 μg/mL) were obtained for children who received LCM monotherapy, and a very impressive proportion of 70.6% was seen in those 17 patients who became seizure free over a minimum 3-month follow-up therapy (Figure 2B; Table 3). Therefore, our data in hands suggest that aiming at a *C*_0_ (2.0–7.0 μg/mL) may be feasible when LCM is used as monotherapy or adjunctive therapy for Chinese children with focal epilepsy.

One of the major strengths of the current study was our ability to monitor the LCM plasma levels and thus we could evaluate the effects of various variables on the dose-adjusted plasma levels (*i.e*., *C*_0_/Dose) of LCM in our study subjects. The demographic characteristics (sex, age and ethnicity) have been identified as the right factors that affect LCM pharmacokinetics (4). Moreover, previous evidence supports the opinion that LCM exposure depends on age and sex, requiring PK monitoring to define the optimal posology that guarantees therapeutic efficacy with tolerable adverse effects (4). In the present study, a combined data from LCM monotherapy and adjunctive therapy in *C*_0_/Dose ratio revealed no significant difference between patients of both sexes (Figures 3D, 4D). Sex had no relevant effects on the LCM *C*_0_/Dose ratio in healthy adults and adults with focal epilepsy (29), but no similar study is available in children up to now.

In our study, of note, the increasing age decreased the *C*_0_/Dose ratio of LCM used either alone or in combination with other ASMs (Figures 3B, 4B), which could be partly explained by an inverse relationship between plasma concentration and systemic clearance. Specifically, the *C*_0_/Dose ratio in patients aged 1– ≤ 6 and 6– ≤ 12 years was significantly higher than those aged 12– ≤ 18 years, by 81 and 29%, respectively, which is in line with a previous report (27). Interestingly, a decrease in *C*_0_/Dose was seen with age indicating an increase in total clearance with age which is in line with the ontogeny of CYP3A4, CYP2C9, and CYP2C19 involved in LCM metabolism.

Also, we have found a lower *C*_0_/Dose ratio in children who have a higher BW. The *C*_0_/Dose ratio in patients with a BW of ≥40 kg was 1.7-fold lower than in patients with a BW of ≤ 20 kg. Similar findings had been reported in our previous study on tacrolimus concentration-to-dose ratio in children with refractory nephrotic syndrome (30). Why older children with higher BW presented lower *C*_0_/Dose ratio of LCM than those younger counterparts could be partly explained by PK characteristics. The preferential distribution of LCM in extracellular fluids implies that total body water determines plasma concentration (4, 31).

Another important finding in the present study was the assessment of potential drug-drug interactions between LCM and other ASMs. Notably, LCM coadministration with OXC, but not LEV or VPA, significantly decreased the *C*_0_/Dose ratio by comparison with LCM monotherapy (*P* = 0.031; Figure 5). This result suggested a potential PK interaction from the perspective of drug action mechanisms. Pratima Gulati et al. (32) previously suggested that the concomitant use of SCBs did not significantly influence response to LCM. In the present study, there was a clear trend that SCB agents may decreased LCM plasma levels (Figure 5). Particularly, OXC substantially lowered LCM plasma levels, which might result in a reduced efficacy of LCM. Thus, a higher LCM dose might be needed for patients taking concomitant OXC, albeit on an individual patient basis. Collectively, our study provided evidence of complex drug-drug interactions between LCM and concomitant ASMs. More research is required for a complete and clear description of the potential drug interactions, reinforcing the importance of LCM concentration monitoring.

However, our study has several limitations due to its retrospective design nature. Firstly, this was a single-center study with a small sample size because of the new approval in China for pediatric patients. Thus, our findings as a reference should be interpreted with caution. Secondly, 76 children were included but they had variable therapy periods and we had to rely on the real-world clinical reporting rather than prospective patient seizure diaries. This prompted us to summarize the effectiveness data of LCM, alone or adjunctive, over different periods with variable numbers of patients. Thirdly, adverse reactions may be underreported due to the data collected sporadically rather than by a structured questionnaire at clinical visits. Nevertheless, the real-world clinical findings in this study for efficacy and safety, especially for LCM plasma monitoring in children, may be very useful for pediatric clinicians and TDM pharmacists when they try to tailor LCM dosages for precision therapy.

## Conclusions

In conclusion, this retrospective study found that LCM treatment used alone or with other ASMs in children with focal epilepsy can reduce the seizure frequency with adverse reactions reported in a minority. We also identified several contributing factors to variable *C*_0_/Dose ratio of LCM in children with epilepsy. Children with higher BW and older age have a lower *C*_0_/Dose ratio. Complex drug interactions between LCM and other concomitant ASMs were revealed. Based on the data in our hands, the reference range, *i.e*., 2.0–7.0 μg/mL, for routine LCM monitoring may be feasible when LCM is taken as a monotherapy or combined with other ASMs in Chinese children with epilepsy.

## Data availability statement

The raw data supporting the conclusions of this article will be made available by the authors, without undue reservation.

## Ethics statement

The studies involving human participants were reviewed and approved by the Ethics Committee of the Children's Hospital of Nanjing Medical University granted the ethical approval for the study (Protocol number 202204021-1). Written informed consent to participate in this study was provided by the participants' legal guardian/next of kin. Written informed consent was obtained from the individual(s), and minor(s)' legal guardian/next of kin, for the publication of any potentially identifiable images or data included in this article.

## Author contributions

YL and H-LG had full access to all the data in the study and take responsibility for the integrity of the data and the accuracy of the data analysis. Concept and design: JC and X-PL. Drafting of the manuscript: YL and FC. Critical revision of the manuscript: FC. Administrative, technical, or material support, and supervision: FC and X-PL. Acquisition, analysis, or interpretation of data, contributed to the article, and approved the submitted version: all authors.

## Funding

This research was supported by the Specially Appointed Medical Expert Project of the Jiangsu Commission of Health (2019), Jiangsu Research Hospital Association for Precision Medication (JY202108), and by the CAAE Epilepsy Research Fund of China Association against Epilepsy (CU-2022-024).

## Conflict of interest

The authors declare that the research was conducted in the absence of any commercial or financial relationships that could be construed as a potential conflict of interest.

## Publisher's note

All claims expressed in this article are solely those of the authors and do not necessarily represent those of their affiliated organizations, or those of the publisher, the editors and the reviewers. Any product that may be evaluated in this article, or claim that may be made by its manufacturer, is not guaranteed or endorsed by the publisher.
